# Pulmonary impairment after tuberculosis and its contribution to TB burden

**DOI:** 10.1186/1471-2458-10-259

**Published:** 2010-05-19

**Authors:** Jotam G Pasipanodya, Scott JN McNabb, Peter Hilsenrath, Sejong Bae, Kristine Lykens, Edgar Vecino, Guadalupe Munguia, Thaddeus L Miller, Gerry Drewyer, Stephen E Weis

**Affiliations:** 1 School of Public Health, University of North Texas Health Science Center at Fort Worth, Fort Worth, Texas, USA; 2 Department of Medicine, University of North Texas Health Science Center at Fort Worth, Fort Worth, Texas, USA; 3 Centers for Disease Control and Prevention, Atlanta, GA, USA; 4 Tarrant County Public Health Department, Fort Worth, Texas, USA; 5 Division of Infectious Diseases, UT Southwestern Medical Center, 5323 Harry Hines Blvd, Dallas, Texas 75390-9113, USA; 6 Eberhardt School of Business and Thomas J. Long School of Pharmacy & Health Sciences, University of the Pacific, Stockton, California, 95211, USA

## Abstract

**Background:**

The health impacts of pulmonary impairment after tuberculosis (TB) treatment have not been included in assessments of TB burden. Therefore, previous global and national TB burden estimates do not reflect the full consequences of surviving TB. We assessed the burden of TB including pulmonary impairment after tuberculosis in Tarrant County, Texas using Disability-adjusted Life Years (DALYs).

**Methods:**

TB burden was calculated for all culture-confirmed TB patients treated at Tarrant County Public Health between January 2005 and December 2006 using identical methods and life tables as the Global Burden of Disease Study. Years of life-lost were calculated as the difference between life expectancy using standardized life tables and age-at-death from TB. Years lived-with-disability were calculated from age and gender-specific TB disease incidence using published disability weights. Non-fatal health impacts of TB were divided into years lived-with-disability-acute and years lived-with-disability-chronic. Years lived-with-disability-acute was defined as TB burden resulting from illness prior to completion of treatment including the burden from treatment-related side effects. Years lived-with-disability-chronic was defined as TB burden from disability resulting from pulmonary impairment after tuberculosis.

**Results:**

There were 224 TB cases in the time period, of these 177 were culture confirmed. These 177 subjects lost a total of 1189 DALYs. Of these 1189 DALYs 23% were from years of life-lost, 2% were from years lived-with-disability-acute and 75% were from years lived-with-disability-chronic.

**Conclusions:**

Our findings demonstrate that the disease burden from TB is greater than previously estimated. Pulmonary impairment after tuberculosis was responsible for the majority of the burden. These data demonstrate that successful TB control efforts may reduce the health burden more than previously recognized.

## Background

Tuberculosis (TB) is an important cause of morbidity and mortality worldwide. In 2003, an estimated 8.8 million new TB cases and 1.7 million TB deaths were reported globally [1,2]. The spread of HIV, a rise in TB drug resistance, and the increase in global movement of persons from high to low TB incidence areas have contributed to the growing health threat due to TB [1-4]. Worldwide there are an unknown but substantial number of patients who have survived acute TB disease.

Pulmonary TB survivors frequently experience structural [5,6] and functional lung sequelae [7,8] that vary in severity that have recently been more completely described [8-10]. Measuring the negative health impacts of these sequelae is an important component to determining the burden of TB, a disease in which control efforts are largely publicly funded. The translation of physical burden into a standard measure of health effect is now possible and allows comparative analyses between alternative uses of funding.

An accurate estimation of disease burden should include all known negative health effects [3,4,11-20,22,23]. This is especially true for TB in low-incidence countries, where death from TB is rare and therefore the TB disease burden may be underappreciated [2,14-16,20,22,23]. The measurement of non-fatal, negative health effects is important to fully estimate TB disease burden [11-14]. Pulmonary impairment after tuberculosis was recently described as a non-fatal negative health effect [8-10]. Pulmonary impairment after TB was identified in more than half of microbiologically cured patients, varied in severity with approximately 10% having lost more than half of their lung function [8]. Pulmonary impairment after tuberculosis has not been incorporated in assessments of TB burden [2,14-16,20,22,23].

One means to describe negative health effects is by using disability adjusted life-years (DALYs). DALYs are numerical values representing both the sum of years-of-life-lost due to premature mortality and the years lived with disability [13]. DALYs represent negative health effects of disease in a population. One DALY represents the loss of one year of equivalent, complete health. Therefore, DALYs can measure gain or loss of population health in a value that is comparable across different resources used for prevention and control.

There are diverse methods for measuring non-fatal health outcomes and as a consequence some evaluations have calculated DALYs in markedly different ways [14,15,19,20]. Prior estimates of DALYs lost from TB have included only health lost from acute illness and death [14-23]. As a result, the global [14] and U.S. national [15] TB burden estimates do not fully reflect the consequences of surviving TB disease. For practical and benchmarking purposes, we used the Global Burden of Disease Study methods on a defined population for a defined period of time to recalculate the relative DALYs associated with the burden of TB but including pulmonary impairment after tuberculosis in the estimate. In addition we compared effects of using the local Texas life-tables in place of the Japanese life tables commonly used in estimating DALYs.

## Methods

We analyzed data of culture-positive TB cases treated at Tarrant County, Texas Public Health (TCPH) TB Elimination Division from January 2005 to December 2006, including Tarrant County TB deaths reported during the same period. TCPH serves approximately 1.7 million residents in north central Texas [24]. By Texas statute all persons with positive TB cultures must be reported to the local health authority, and by policy of the Texas Department of State Health Services all persons with TB are treated with directly observed therapy (DOT) [25,26].

### Overall TB Burden (DALY)

TB burden was calculated for 16 TB deaths and 161 culture-confirmed TB patients treated at TCPH between January 2005 and December 2006 using identical methods and life tables as the Global Burden of Disease Study by Murray et al [13,26]. Years-of-life-lost from premature mortality (YLL) and Years-lived-with-disability (YLD) were estimated with respect to a standard expectation of life at each age using West 26 model life tables [13,14,27,28]. We used a hypothetical norm of the maximum possible life expectancies for all ages to make DALYs comparable across cultures, countries, and regions [13,14]. With this assumption, Japanese life expectancies at birth where females are expected to live for 82.5 years were used [13,14,27,28] with a Texas life-table used for comparison [29]. Clinical TB includes in addition to culture negative tuberculosis diseases other than tuberculosis. To improve precision of TB burden estimates we excluded clinical TB from our calculations.

### Disability data-Years lived with disability (YLD)

Non-fatal health impacts of TB were divided into years lived with both acute (YLD-acute) and chronic disability (YLD-chronic). Years lived-with-disability therefore combines duration and severity of acute disease and disability from sequelae. Years lived-with-disability-acute (YLD-acute) was defined as TB burden resulting from illness prior to completion of treatment and the burden from treatment-related side effects. It included disability from tuberculosis at diagnosis to end-of-treatment. The duration of YLD-acute was assumed to be 6-months [16-20]. YLD-acute was calculated by multiplying the duration of acute disease by an age-weighted disability weight factor, discounted at 3% per year [13,17,18,30]. Years lived-with-disability-chronic (YLD-chronic) was defined as permanent TB burden from disability resulting from PIAT. YLD-chronic was calculated by multiplying the estimated remaining years of life after completion of therapy by age-weighted disability weight factor, also discounted at 3%, per year, for the duration of remaining years of life lived with disability. Further details about DALY computation assumptions are shown in the attached appendix.

Disability weight, a scaling factor that ranges from 0 (representing the best possible health state) to 1 (representing the worst possible living heath state; equated to death), is an essential component of years lived with disability computation. The disability weights used were obtained from prior reports of the Global Diseases Study and the Dutch disability weighting study [14,16,18]. They were 0.294 for ages 0-14, 0.264 for 15-59 and 0.274 for the 60+. We also derived disability weights stratified by impairment levels from these TB survivors using the St George's respiratory questionnaire and Spirometry [8,9]. The disability weights for non-impaired persons were 0.173. Those for mildly impaired was 0.248, moderately impaired 0.273 and severely impaired 0.377 [9]. We tested these locally derived disability weights in sensitivity analysis. Extra-pulmonary TB cases were excluded in estimating YLD-chronic estimates because there are no data to support assumptions for burden from long-term sequale.

### Mortality data-Years of life lost (YLL)

Years of life-lost were calculated for each reported death due to TB as the difference between life expectancy and age-at-death. These differences were then summed across the Tarrant County population using the identical methods and life tables as the Global Burden of Disease Study [13,14]. Each death due to TB was attributed to a single cause according to the International Classification of Disease (ICD) for attribution of causes of death [31]. Years-of-life-lost was then computed using the formula *YLL[r, K, β] = KCe^ra^/(r+ β)^2^{e^-(r+ β)(L+a)^[-(r+ β)(L+a)-1]-e^-(r+ β)a^[-(r+ β)a-1]}+(1-K)/r(1-e^-rl^*) where K = age weighting modulation factor; C = Constant; r = discount rate; a = age of death; β = parameter from the age weighting function; and L = standard expectation of life at age-of-death [13,14]. The value of C = 0.01458 together with β = 0.04 and K = 0 produce uniform age weighting when the discount rate is zero. Discounting is the standard method used to adjust the value of money, cost, and benefits to reflect preference for present rather than future utility; interest is paid on monetary loans for the same reason [4,13-20].

### Sensitivity Analysis

The Global Burden of Disease Study of 1996 methods and assumptions were used to estimate Tarrant County data that included disability due to pulmonary impairment after tuberculosis. This base DALY calculation was made for ease of comparison. Since years lived-with-disability-acute contributed very little to total burden, it was not included in sensitivity analysis. We assumed survivors of TB treatment have a normal life expectancy [13,14,17]. Total DALYs were stratified by gender, age and race. DALYs were not adjusted for co-morbidity. Mortality included all TB deaths reported in Tarrant County. The base DALY *[r, K, β] *was; r = 0.03, K = 0 and *β = 0.04*. Prior data suggests that estimates of TB burden should vary based on severity of impairment resulting from TB disease, in addition to the age at which disease occurs [8], [9], [13-20], Vecino ME, Pasipanodya JG, Slocum PC, Bae S, Munguia G, Miller TL, Drewyer G, Weis SE. 2010]. Evidence for chronic lung impairment in patients cured of tuberculosis; Submitted]. Sensitivity analyses were performed with varied discount rates, disability weights, age weights, and local life tables [8,9,13,14,16,32]. Discount rates varied from 0% to 10% and the disability weights from 0.173 to 0.377 depending on disease severity. We also used previously published data (figure sixteen.one from reference 33) to test potential policy implications of these data on TB interventions.

Parametric and non-parametric tests were performed and reported unless otherwise stated. Data were analyzed using SPSS version 12 for Windows (SPSS Inc. 233 S. Wacker Dr, Chicago IL 60606-6307) and Microsoft Excel 2002 (Microsoft Corporation, Richmond, WA).

## Results

### Descriptive

There were 224 TB cases in Tarrant County, Texas, between January 1, 2005 and December 31, 2006. Forty-seven patients (22 females) were treated for clinical TB and none of these died. Clinical TB was excluded from further analysis henceforth. Of the remaining 177 patients with tuberculosis confirmed by culture or histology 155 (88%) had pulmonary and 22 (12%) extra-pulmonary tuberculosis. During this period 16 (9%) patients died and of these 14 had pulmonary and 2 had extra-pulmonary TB. Male TB patients outnumbered female (123 [69%] vs. 54 [31%]). The median age in years of males (interquartile range) was 47 (23) versus 39 (24) for females. TB patients were racially heterogeneous with Caucasians and Hispanics together comprising 50%, while the HIV prevalence was 11% (table 1).

**Table 1:** Demographic characteristics of patients with culture confirmed tuberculosis between January 1, 2005 and December 31, 2006, Tarrant County, Texas.

Demographic Characteristic		Male	Female	Total
		N (%)	N (%)	N (%)
Mortality Status	Alive	109 (89)	52 (96)	161 (91)
	Dead	14 (11)	2 (4)	16 (9)
Disease Site	Pulmonary	113 (92)	42 (78)	155 (88)
	Extra-pulmonary	10 (8)	12 (22)	22 (12)
Age groups	0-4	2 (2)	5 (9)	7 (4)
	5-14	0	0	0
	15-29	17 (14)	10 (19)	27 (15)
	30-44	33 (27)	20 (37)	53 (30)
	45-59	43 (35)	11 (20)	54 (31)
	60-69	15 (12)	4 (7)	19 (11)
	70-79	10 (8)	3 (6)	13 (7)
	80+	3 (2)	1 (2)	4 (2)
Racial Groups	Caucasian	33 (27)	11 (20)	44 (25)
	Hispanic	32 (26)	13 (24)	45 (25)
	African-American	31 (25)	10 (18)	41 (23)
	Asians, Africans, Indians, Others	27 (22)	20 (37)	47 (27)
HIV^# ^status	Negative	99 (80)	47 (87)	146 (83)
	Positive	17 (14)	3 (6)	20 (11)
	Unknown	7 (6)	4 (7)	11 (6)
Country of Birth	USA born	67 (55)	21 (39)	88 (50)
	Foreign-born	56 (45)	33 (61)	89 (50)
**Total**		**123 (100)**	**54 (100)**	**177 (100)**

#HIV = Human Immunodeficiency Virus

### Overall TB burden - DALY

**Figure 1:**
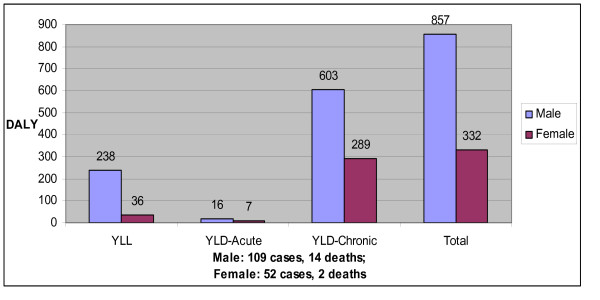
**Years of-life-lost (YLL), Years lived-with-disability-acute (YLD-Acute), Years lived-with-disability-chronic (YLD-Chronic) and Total Disability-Adjusted Life Years (DALY), for males and females**. Total = YLL + YLD-Acute + YLD-Chronic.

A total of 1189 DALYs were lost among these 177 TB patients, with 274 DALYs lost from years-of-life-lost and 915 DALYs lost from acute and chronic years lived-with-disability (table 2; figure 1). Males lost nearly 857 DALYs, 72% of the total (figure 1). DALY loss varied significantly by age (p < 0.001): persons 30-44 years of age had a total of 407 (34% of total) DALYs lost. Most (75%) of the DALYs loss in the 30-44 age group were due to years lived-with-disability-chronic. Years-of-life-lost accounted for 23% of the DALYs lost (table 2). Among African-American patients, 18% of the total DALYs lost was due to years-of-life-lost in patients younger than 60 years of age (table 2). Caucasians comprised 25% of the patient population and contributed 25% of total DALYs lost. The 22 (12%) patients with extra-pulmonary TB lost only 39 DALYs (3% of total). The 16 TB deaths included 14 males and 2 females; 4 Caucasians, 4 African-American, 3 Hispanics, 2 Asians and 1 African. The majority of years-of-life-lost (70%) resulted from deaths in persons younger than 60 years of age. US born persons contributed two thirds of total years-of-life-lost (table 2).

**Table 2:** The mean and total TB burden of 161 survivors and 16 patients who died of Tuberculosis between January 2005 and December 2006 Tarrant County

				Years of Life Lost		Years of Life-Lived with Disability-acute		Years of Life-Lived with Disability - chronic (PIAT)		Disability Adjusted Life Years	
		Alive	Dead	Mean	Sum	Mean	Sum	Mean	Sum	Mean	Sum
Race	Caucasian	40	4	1.48	65.25	0.13	5.75	5.19	228.34	6.80	299.20
	Hispanic	42	3	1.03	46.50	0.13	6.04	5.82	261.80	6.99	314.34
	African-American	37	4	1.78	72.92	0.13	5.32	4.74	194.33	6.65	272.57
	AAIO*	42	5	1.90	89.25	0.13	6.03	4.42	207.61	6.44	302.90
Age groups	0-4	7	0	0	0	0.14	1.01	7.65	53.55	7.79	54.56
	5-14	0	0	0	0	0	0	0	0	0	0
	15-29	27	0	0	0	0.14	3.88	6.63	179.04	6.77	182.92
	30-44	49	4	1.81	96	0.13	7.04	5.74	304.55	7.69	407.45
	45-59	49	5	1.72	93	0.13	7.04	4.99	269.5	6.84	369.54
	60-69	13	6	4.15	78.92	0.13	1.87	2.82	53.58	7.07	134.37
	70-79	13	0	0	0	0.14	1.87	2.11	27.47	2.26	29.34
	80+	3	1	1.5	6	0.11	0.43	1.1	4.4	2.71	10.83
Disease Site	Pulmonary	141	14	1.69	261.67	0.13	20.13	5.6	868.59	7.42	1150.38
	Extra-pulmonary	20	2	1.63	35.75	0.13	2.88	N/A*	N/A*	1.76	38.63
Country of Birth	USA born	78	10	2.27	199.67	0.13	11.07	4.65	409.05	7.04	619.79
	Foreign-born	83	6	1.1	97.75	0.13	11.93	5.16	459.54	6.4	569.22
HIV status	Negative	139	7	1.13	164.5	0.14	19.84	4.98	726.53	6.24	910.87
	Positive	17	3	2.71	54.17	0.12	2.44	5.4	107.93	8.23	164.54
	Unknown	5	6	7.16	78.75	0.07	0.72	3.1	34.13	10.33	113.6
**Total**		**161**	**16**	**1.55**	**273.92**	**0.13**	**23.14**	**5.04**	**892.09**	**6.72**	**1189.01**

*N/A Not Applicable; AAIO Africans, Asians, Indians & Other racial groups

**Figure 2:**
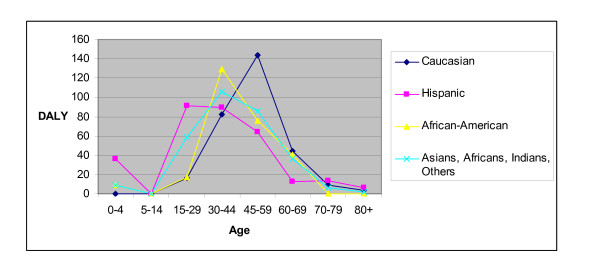
**Racial Distribution of TB burden stratified by age groups for the 177 culture confirmed Tuberculosis patients seen between January 1, 2005 and December 31, 2006, Tarrant County, Texas**.

There were differences in DALY loss and its sources between Caucasians and other races (table 2; figure 2). There was no tuberculosis or correspondent DALY loss in Caucasian children under 5 years old. There was substantial DALY loss to young children in other ethnic groups. Eleven percent of the DALY loss in Hispanics occurred in children younger than 5 years (table 2). Three percent of DALY loss in each of African-American and all other races as a group occurred in children less than 5 years old. None of the DALY loss to Caucasians between age 5 and 45 years was due to years-of-life-lost premature mortality, but years-of-life-lost substantially contributed to total DALY loss in non-Whites younger than 45 years. Premature mortality in those under age 45 accounted for 31% (48.5/155.7) of DALYs lost to African-Americans, 11% (23.3/217.08) to Hispanics, and 14% (24/173.57) to combined other races. Race was not a significant predictor (p = 0.273) for total DALY when age was controlled for; but was statistically significant (p = 0.028) in predicting YLD-chronic (data not shown).

### Disability data-Years lived with disability (YLD)

YLD comprised the majority (75%) of the TB burden. YLD-acute contributed 23 (2%) of the total DALYs lost, and YLD-chronic contributed 235 (73%) (table 2; figure 1). The mean YLD lost-per-patient for YLD-acute was 0.14 and for YLD-chronic was 5.04 DALYs. The overall burden of TB was greater among males since more males had and died with TB. However, the median value for YLD-acute, YLD-chronic, and total DALY for men was not significantly different from females (*p-*values 0.102, 0.066 and 0.575, respectively). YLD-chronic was greater in foreign-born than US-born patients, as foreign-born subjects were younger when they developed tuberculosis (table 2).

### Mortality data-Years of life lost (YLL)

The mean TB burden from years-of-life-lost was 1.55 DALYs per patient. The tuberculosis burden from years-of-life-lost was greater in US than foreign-born persons but did not reach statistical significance (2.27 vs. 1.10 DALYs) (table 2).

### Sensitivity Analysis

**Figure 3:**
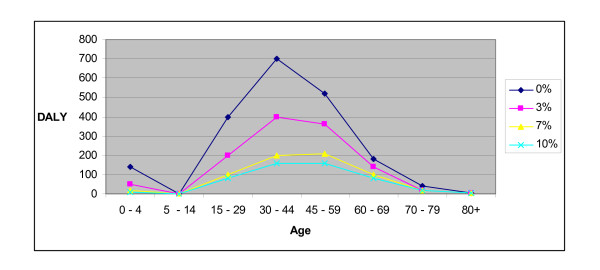
Sensitivity analysis: comparisons of the effects of varying the discount rates from 0 to 10%, on total TB Burden in DALY*. *Uniform age weights were applied throughout

We also analyzed the effects of varying the discount rates (figure 2) and applying full age weights (data not shown). Changes in the discount rate from 0% to 10% had little effect (5% changes) on the distribution of burden by gender, while a lower discount rate increased and clustered the DALY burden at lower age groups (figure 3). Applying full age weights lowered the total TB burden by 6% (figure 3). When we used disability weights that adjusted for clinical severity of disease and were obtained from the same patient population [9,10], total burden was 45% lower compared to an analysis that used fixed disability weights (data not shown).

**Figure 4:**
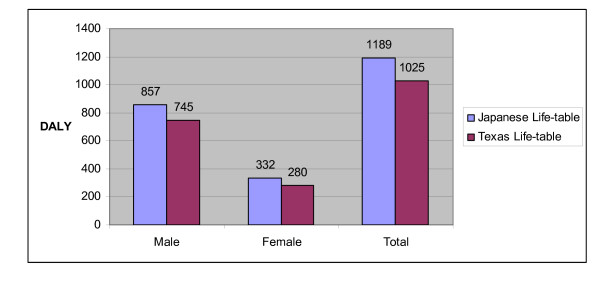
**Comparison of the effects of using Texas life-tables instead of the Japanese life-tables on total tuberculosis burden**.

When Texas life-tables were used to compute TB burden, the overall TB was lower 1025 DALYs compared to 1189 DALYs when Japanese life-tables were used. Males contributed 745 DALYs and females 280 DALYs. A uniform 14% difference in tuberculosis burden applicable across gender and age-groups was observed (figure 4).

## Discussion

Prior estimates of DALYs lost from TB included negative health effects from YLD-acute, and years-of-life-lost, but did not include YLD-chronic [2,14-16,20,22,23]. Using methods recommend by the World Bank and used by Murray et al in the Global Burden of Disease Study we estimated the burden of TB including YLD-chronic. In the urban area of Tarrant County, Texas, with reported 0.06/1000 incidence of TB [2], we calculated the burden to be 0.37 DALYs lost /1000 population, per year. Prior estimates of the TB burden in areas with similar TB incidence, using the same methods but only measuring YLD-acute and years-of-life-lost were 0.07 DALYs lost /1000 population, per year [3,16,20]. Our data from this study show that disability is a major component of TB burden, and that prior estimates of the DALYs lost from TB accounted for only 25% of the total loss (table 2; figure 1).

Each occurrence of pulmonary TB illness results in both YLD-acute and YLD-chronic from pulmonary sequelae. We found that only 2% of the TB burden resulting was due to acute disease. This YLD-acute estimate was similar to previously reported WHO estimates of TB burden (approximately 0.1 DALY per case for areas with very low adult and child mortality) [16,20].

Prior estimates of DALYs lost to TB do not consider pulmonary impairment after tuberculosis. Pulmonary impairment after tuberculosis is a common, life-long condition [8-10], Vecino ME, Pasipanodya JG, Slocum PC, Bae S, Munguia G, Miller TL, Drewyer G, Weis SE. 2010]. Evidence for chronic lung impairment in patients cured of tuberculosis; Submitted]. Nearly 60% of patients have measurable impairment after microbiological cure that ranges from mild impairment to severe disability [8-10]. Exclusion of YLD-chronic from previous TB burden estimates has led to recommendations that are incompletely informed, namely that when TB incidence is stable or declining in a population, passive intervention is more cost-effective than active case finding or treatment of latent TB infection [4,33-35]. Such arguments support reduced resource allocation for TB programs and that policy action may have contributed to resurgence in U.S. TB incidences seen in the early 1990s. Exclusion of PIAT in estimates of TB burden undervalues the cost-effectiveness of TB prevention activities leading to inadequate resources allocated to prevention [2].

Mortality due to TB is an important component of the overall burden [1-5,14]. Years-of-life-lost accounted for 1.55 of the total 6.72 DALY lost per TB patient (table 2). Previous estimates of the TB burden from low-incidence areas reported 0.85 DALY lost from years-of-life-lost per TB patient [16,20]. We believe the higher years-of-life-lost found in this analysis was due to our study population including only culture-confirmed TB. Clinical cases of TB are less likely to suffer mortality and their inclusion would have lowered years-of-life-lost [5]. The relationship between illness-related mortality and disability is often expressed as YLD: YLL ratio and has been used to estimate DALYs lost from TB [19,21]. This ratio can be used to estimate disability from an illness in a community from mortality statistics. We found the YLD: YLL ratio for TB to be 3.34. When this ratio was calculated for TB without including YLD-chronic, the ratio was 0.08. Not including YLD-chronic would have resulted in YLD: YLL ratios that would have underestimated the TB burden. Additionally these data demonstrated that in low-incidence countries TB causes more DALYs lost from disability than death.

The use of DALY to assess TB burden highlights previously recognized TB racial disparities that are less apparent when either notification or mortality rates alone are used [2]. Figure 3 illustrating the TB burden in the <5 years age group is consistent with recent TB transmission to children among non-Caucasians. Substantial health loss occurred to other racial groups at earlier ages than to Caucasians in our cohort, a disparity that has social and other implications, and indicates that practices to reduce transmission or to prevent mortality may yield disproportionate benefits to these populations. Ranking burden of disease by DALY loss gives information beyond the usual disease descriptors of incidence, mortality, and age at illness. As an illustration of the added information from using DALY to calculate disease burden is aseptic meningitis. Aseptic meningitis is far more common in Tarrant County than TB. However mortality and long-term sequelae are extremely rare. Therefore the disease burden measured in DALY loss from aseptic meningitis is much less than that due to tuberculosis. Without combining the disease incidence, mortality, and impairment into a single number, it is difficult to compare the respective disease burdens from the two diseases.

There are emerging technologies for diagnosis and treatment of TB that are close to clinical implementation. These include the use of gamma interferon release assays to diagnose LTBI, isoniazid and rifapentine to shorten treatment of LTBI, and moxifloxacin containing regimes to shorten treatment of active TB [36]. These data suggests that the greatest health savings may be achieved through strategies to prevent TB rather than strategies to shorten its treatment.

This study gives insight into their potential effect on health lost from TB. Interventions that result in more persons completing LTBI therapy will prevent 6.72 DALY per case of TB averted. In contrast, interventions for shortening treatment of TB would result in little DALYs saved. Reducing TB treatment duration by 50% would have minimal effect on TB burden, as it would save <0.02 DALYs per patient. In addition shorter TB treatment duration, assuming current costs of between US$5 and US$350 per DALY gained, would not reduce the chronic pulmonary impairment associated with TB [8-10,33]. If preventing pulmonary impairment after tuberculosis is considered, costs of current standard latent tuberculosis infection therapy (daily isoniazid for 9 months) falls to under US$2500 per DALY gained. The benefit of treating latent tuberculosis infection becomes comparable to those of treating non-infectious TB [33].

The use of discounting in economic health evaluations and appropriate discount rates are controversial [13,18,32,37-39]. We analyzed our results using the discount rate recommend by the CDC and the U.S Preventive Services Task Force (USPSTF) of 3% [13,14,32]. Irrespective of the discount rates used the present value of DALYs lost to the cohort was significantly greater than previous estimates. To improve the validity and precision of the TB burden estimates, we tested disability weights derived directly from the same TB-afflicted population in sensitivity analysis [9]. Using these locally derived disability weights did not change our conclusions. This analysis indicates that the increased TB burden identified in this study is independent of the discount rates or disability weight used.

There are limitations to these estimates. We did not adjust for co-morbidity including relapses, re-infections drug-resistance, or acquired immuno-deficiency syndrome (AIDS). Additionally these results should be considered a conservative measure of tuberculosis burden, as they do not include the contribution of clinical or extra pulmonary tuberculosis or possible excess mortality after cure. In addition, current DALY computation does not weigh-in the effects of epidemiological parameters other than age and gender. Pediatric and adolescent TB are infrequent in Tarrant County, as in other low-incidence areas; we were therefore unable to adequately test the effects of age or the interaction of age and ethnicity in the final analysis.

Even though the DALY is widely used and possibly one of the best ways to quantify and estimate measurement of morbidity and mortality for a given disease in a population; there has been controversy over the appropriateness of its use in the past especially when applied to certain disadvantaged communities [30]. For example, DALY assumptions are limited when applied to societies that have clearly different life-tables from Japan [14,28,30]. We found a 14% difference in tuberculosis burden when DALY was calculated using Texas life tables (figure 4). For comparison of local burden disease, certainly use of local life tables would account for this important difference. One of the aims of this study was comparison of tuberculosis burden with or without inclusion of PIAT using prior established methods. When these data are combined PIAT contributed significantly to overall tuberculosis burden. Differential age weights would increase importance of pediatric and adolescent mortality [14,21]. While the results are from a single geographic area we included consecutive subjects within the defined period to reduce selection bias and the population was heterogeneous. As a result we feel that despite these limitations the results are representative of the TB burden in similar populations.

## Conclusions

Using the Global Burden of Disease Study methods we demonstarate that pulmonary impairment after TB contributes importantly to the TB burden, which is greater than previously estimated. These data suggests that in low-incidence countries successful strategies to prevent TB from developing may prevent more health burden than previously recognized.

## Competing interests

Potential conflicts of interest: JGP - none, EV - none, GM - none, TLM -none, SJNM-none, PH- none, SB -none, GD -none, SEW -none.

## Authors' contributions

Conception and designing of the study was done by JGP, SJNM, PH, KL, GD, and SEW.

EV, GM, TLM, GD and SEW collected the data, while JGP, SB, KL and SEW analyzed the data. All authors wrote the manuscript. All authors read and approved the final manuscript.

## Appendix: Glossary of terms and acronyms used

### Pulmonary Impairment After Tuberculosis (PIAT)

Pulmonary impairment after tuberculosis (PIAT) refers to chronic pulmonary function loss that occurs in persons who have achieved microbiologic cure of pulmonary tuberculosis. Levels of impairment were determined in previous studies via spirometry using American Medical Association's Guide to Evaluations on Permanent Impairment (fifth edition) [40]. Impairment was scaled none, mild, moderate, or severe as follows: none (FVC > 80% predicted and FEV_1 _> 80% predicted), mild (FVC 60-79% predicted and FEV1 60-70% predicted), moderate (FVC 51-59% predicted, FEV_1 _41-59% predicted) and severe (FVC < 50% predicted, FEV_1 _< 40% predicted) [8,40]. Disability associated with PIAT was evaluated by use of a validated health related quality of life instrument, the St George's respiratory questionnaire [9].

### Disability adjusted life years (DALY)

DALY represents negative health effects of disease in a population. One DALY represents the loss of one full year of equivalent, complete health. DALYs were obtained from the addition of two components: years of-life-lost (YLL) and years lived-with-disability (YLD). Thus DALY = YLL + YLD. To be consistent with Global Burden of Disease Study by Murray et al we used a hypothetical norm of the maximum possible life expectancies for all ages [13,14]. This makes DALY comparable across cultures, countries, and regions. With this assumption Japanese life expectancies at birth of 82.5 years for females and 80.5 years for males were used [13,14,26-29]. DALY measurement using this standard is based on the egalitarian principle that allows death at the same age to contribute equally to burden of disease in different communities across the globe.

### Years of-life-lost (YLL)

YLL was calculated by defining a life expectancy and then subtracting the actual age at death for each subject who died.

### Years lived-with-disability (YLD)

YLD are the non-fatal outcomes of TB. For this analysis non-fatal, health impacts of TB were divided into years lived-with-disability-acute and years lived-with-disability-chronic. The measurements of these non-fatal outcomes of tuberculosis were based on previous work that directly measured pulmonary impairment and disability after tuberculosis [8], [9], Vecino ME, Pasipanodya JG, Slocum PC, Bae S, Munguia G, Miller TL, Drewyer G, Weis SE. 2010. Evidence for chronic lung impairment in patients cured of tuberculosis; Submitted].

### Years lived-with-disability-acute (YLD-acute)

YLD-acute was defined as TB burden resulting from illness prior to completion of treatment and burden from treatment-related side effects. The duration of YLD-acute was assumed to be 6-months to be consistent with Global Disease Study.

### Years lived-with-disability-chronic (YLD-chronic)

YLD-chronic was defined as TB burden from disability resulting from PIAT. The duration of this impairment was assumed to be the time from completion of TB treatment to the predicted life expectancy.

**Discounting **in this article refers to time preference for consumption; when given a choice people generally value healthy life in the present more than potential healthy life in the future. Therefore potential life lost in the future is valued less and is usually discounted at 3%.

### Age weights

In DALY calculations, the age-weighting function specifies the relative value of life lived at different ages. It is used in the measurement of years-of-life-lost and years lived-with-disability. Age weighting formula is Cxe^-*βx*^, where x is the age corresponding to each year of life lost; C = 0.1658; e is the natural logarithm which is a constant approximately equal to 2.7183, β = 0.04.

This function can be can be adjusted by introducing a constant (K) in order to modify weights, y = K Cxe^-βx ^+ (1-K). K is the age weighting modulation factor, a parameter that allows uniform (K = 0) or nonuniform (K = 1) age-weighting to be used. When K = 0, years lost have equal value. When K has a value higher than 0, years lost acquire different values depending on age. The age weight increases gradually from birth to the age of 25 and then decreases. Age weights are controversial with strong arguments for and against their inclusion in computation of DALY. Note that YLL = 1/r(1-e^-rl^) in our base case when K = 0.

### Disability weights

The negative health impact of a medical condition can be quantified over the duration of the condition using disability weighting. Disability weights have been calculated in many ways. These include self-assessed with rating scales (such as the visual analogue scales), magnitude estimation (asking direct questions about the relative value of time spent in each health state compared to another) and trade-off methods (such as time trade-off, willingness-to-pay and person trade-off) [16-20]. Disability weights from PIAT in this study were also directly derived from the St George's respiratory questionnaire [9].

### YLD: YLL ratio

The YLD: YLL ratio is years lived with disability (YLD) to years of life lost (YLL). The YLD: YLL ratio can be used to estimate disability from mortality of the disease in communities where morbidity data is generally scarce. The Global Burden of Disease study and the World Health Organization have already established YLD: YLL ratios for various diseases that have been stratified by age group and geographical region. These established YLD: YLL ratios have been used by some developing countries to estimate TB DALYs [19].

## Pre-publication history

The pre-publication history for this paper can be accessed here:

http://www.biomedcentral.com/1471-2458/10/259/prepub
